# Retraction: A DNA inducing VLP vaccine designed for HIV and tested in mice

**DOI:** 10.1371/journal.pone.0203635

**Published:** 2018-08-31

**Authors:** The PLOS ONE Editors

After publication of this article, Cesar Boggiano and Ross Lindsay requested their removal from the author list, noting that they had not agreed to be listed in this article’s byline, and that the article had been submitted and published without their explicit approval. Outdated email addresses were provided for these authors at the time of submission and so they did not receive notifications from the journal office.

In reviewing this case, we identified several concerns about the published work, including that the methods were not sufficiently well-reported to meet *PLOS ONE*’s requirements, the original data were not provided with the article contrary to the Data Availability statement, and questions about the quality and integrity of the Western blot data in Fig 2.

We raised the concerns with the first author, who clarified several aspects of the materials and methods reporting, provided some raw data files, and explained that the original raw data underlying Fig 2 and the Week 5 results in Fig 4 were no longer available. He also did not provide the ICS data underlying the FACS results in Fig 5.

We evaluated the author’s responses in collaboration with a member of *PLOS ONE*’s Editorial Board. The consulted Academic Editor advised that additional molecular analyses would be needed to support the results and conclusions in this article; there was a discrepancy between data file labels and the Methods section as to whether the FACS experiments were conducted at Week 5 or Week 9; and the authors had not included enough experimental replicates to demonstrate the reproducibility of some results.

In light of the different concerns identified and in the absence of the required primary data to substantiate the conclusions of the study, the *PLOS ONE* Editors consider that the findings in this article are not reliable and hence we are issuing a retraction.

CB and RL agreed with the retraction. AC did not respond.

## References

[1] Calazans A, Boggiano C, Lindsay R (2017) A DNA inducing VLP vaccine designed for HIV and tested in mice. PLoS ONE 12(8): e0183803 10.1371/journal.pone.0183803 28837706 PMC5570355

